# Benchmarking of de novo assembly algorithms for Nanopore data reveals optimal performance of OLC approaches

**DOI:** 10.1186/s12864-016-2895-8

**Published:** 2016-08-22

**Authors:** Yesesri Cherukuri, Sarath Chandra Janga

**Affiliations:** 1 Department of Bio Health Informatics, School of Informatics and Computing, Indiana University Purdue University, 719 Indiana Ave Ste 319, Walker Plaza Building, Indianapolis, IA 46202 USA; 2 Centre for Computational Biology and Bioinformatics, Indiana University School of Medicine, 5021 Health Information and Translational Sciences (HITS), 410 West 10th Street, Indianapolis, IA 46202 USA; 3 Department of Medical and Molecular Genetics, Indiana University School of Medicine, Medical Research and Library Building, 975 West Walnut Street, Indianapolis, IA 46202 USA

**Keywords:** Contigs, De novo assembly, De Bruijn, Greedy Extension graph, MinION^®^, Nanopore, N50, Oxford Nanopore

## Abstract

**Background:**

Improved DNA sequencing methods have transformed the field of genomics over the last decade. This has become possible due to the development of inexpensive short read sequencing technologies which have now resulted in three generations of sequencing platforms. More recently, a new fourth generation of Nanopore based single molecule sequencing technology, was developed based on MinION^®^ sequencer which is portable, inexpensive and fast. It is capable of generating reads of length greater than 100 kb. Though it has many specific advantages, the two major limitations of the MinION reads are high error rates and the need for the development of downstream pipelines. The algorithms for error correction have already emerged, while development of pipelines is still at nascent stage.

**Results:**

In this study, we benchmarked available assembler algorithms to find an appropriate framework that can efficiently assemble Nanopore sequenced reads. To address this, we employed genome-scale Nanopore sequenced datasets available for *E. coli* and yeast genomes respectively. In order to comprehensively evaluate multiple algorithmic frameworks, we included assemblers based on de Bruijn graphs (Velvet and ABySS), Overlap Layout Consensus (OLC) (Celera) and Greedy extension (SSAKE) approaches. We analyzed the quality, accuracy of the assemblies as well as the computational performance of each of the assemblers included in our benchmark. Our analysis unveiled that OLC-based algorithm, Celera, could generate a high quality assembly with ten times higher N50 & mean contig values as well as one-fifth the number of total number of contigs compared to other tools. Celera was also found to exhibit an average genome coverage of 12 % in *E. coli* dataset and 70 % in Yeast dataset as well as relatively lesser run times. In contrast, de Bruijn graph based assemblers Velvet and ABySS generated the assemblies of moderate quality, in less time when there is no limitation on the memory allocation, while greedy extension based algorithm SSAKE generated an assembly of very poor quality but with genome coverage of 90 % on yeast dataset.

**Conclusion:**

OLC can be considered as a favorable algorithmic framework for the development of assembler tools for Nanopore-based data, followed by de Bruijn based algorithms as they consume relatively less or similar run times as OLC-based algorithms for generating assembly, irrespective of the memory allocated for the task. However, few improvements must be made to the existing de Bruijn implementations in order to generate an assembly with reasonable quality. Our findings should help in stimulating the development of novel assemblers for handling Nanopore sequence data.

**Electronic supplementary material:**

The online version of this article (doi:10.1186/s12864-016-2895-8) contains supplementary material, which is available to authorized users.

## Background

In recent years, next generation sequencing technologies have been evolving rapidly with the potential to accelerate the research in sequencing biology [1–3]. However, today’s next generation sequencing technologies such as Illumina, 454 Roche, Ion Torrent, SMRT (single –molecule real time sequencing) from Pacific biosciences, have various significant limitations [4] especially amplification biases, short read lengths and genome assembly complexities. For example, Illumina – one of the most commonly used technologies for sequencing in recent years, produces read length of 75–100 base pairs (bp) [5] and hence is believed to suffer from short read lengths resulting in poor assembly of complex regions like long repeats and duplications [4, 6, 7]. However, the application of the SMRT platform to small microbial as well as complex eukaryotic genomes have improved the quality of genome assembly but the commercial availability and price of sequencing are the major limitations of this approach [8–10]. Similar improvements were also accomplished by the Illumina Truseq synthetic long-read sequencing strategy [11, 12], but the long range polymerase chain reaction step included in the library preparation will be a limitation in time-constrained projects, thus making it inaccessible to the whole research community. To overcome such limitations efforts are now been made to develop an inexpensive single-molecule Nanopore-based fourth generation DNA sequencing technology [13–17]. In fact, the concept of single molecule sequencing using biological Nanopores was first proposed by Deamer & Akeson [6, 14, 18] in the year 1996, since then intense efforts have been made to overcome the formidable technical challenges and finally in the year 2014, Oxford Nanopore Technologies Ltd released the first commercial Nanopore sequencer [19, 20] to early access customers. The MinION^®^ device, which is no larger than a typical smartphone consists of pores embedded into a membrane which is placed over an electric grid, as the DNA bases i.e. A(adenine), T(thymine), G(guanine) & C(cytosine) pass through the pores they generate a particular intensity of ionic current, which are further base called using *metricorr* software [21, 22]. The reads generated by this sequencer, can be classified into three types: 2D reads, template reads and complement reads [23]. In our study, we analyzed all three types of reads but mainly focussed on 2D reads since they are optimal reads that consist of consensus information of both the strands [22]. However, similar results were observed upon analyzing all three types of reads, illustrating the reproducibility of our results irrespective of the type of reads analyzed.

Despite the high error content of the MinION reads [20, 24], Aston et al. [25] have demonstrated the utility of these reads in microbial sequencing, which incited the need for the development of new tools either to correct the erroneous reads or for the downstream analysis. The error correcting algorithms have already emerged [24, 26] while, development of downstream pipelines is at nascent stage. A major computational step in any of the DNA sequencing pipelines is assembly and can be defined as a hierarchical data structure that maps the sequence data for the reconstruction of the target genome. This process involves initially grouping the reads into contigs and then contigs into scaffolds thereby generating the assembly. Currently, the most common algorithmic frameworks on which assembly algorithms are developed include the Overlap Layout Consensus (OLC) [27], de Bruijn Graph (DBG) [28] which uses some form of k-mer graph method and greedy extension graphs which use either OLC or DBG [29]. There are about 24 academically available de novo assemblers [29] which have been developed by implementing one of these three assembler algorithms. Most of the assembler algorithms, generally take a file of sequence reads and a quality-score file as input, but for Nanopore data, the quality scores are not available so we failed to test assemblers which insist on the requirement of the quality score file as a compulsory input. An example of one such assembler is PCAP, which although is specifically developed for long read data does not accept reads without quality score information [30]. On the other hand, most of the assemblers such as Newbler failed to assemble Nanopore reads due to the length of the reads. Due to these constraints we finally employed in our study one or two assemblers for each type of assembly algorithm and analyzed the quality, accuracy and efficiency of each assembler on whole genome Nanopore sequencing data for *E. coli* and yeast. Our study unveiled OLC as the optimal algorithm, in multiple contexts benchmarked in this study, providing a direction for further development of assembly tools for Nanopore data.

## Methods

### Data retrieval

Through an early access program of Nanopore sequencer (MAP), Quick et al. [23] sequenced the genome of the model organism, *Escherichia coli K12* substr. Initially, we have used this dataset to benchmark various assembler algorithms but due to high error rate all the existing assemblers failed to assemble such long erroneous reads (5000 – 50,000 bp) (~35 % error) [24]. Later, in January 2015, Schatz and co-workers, developed a novel hybrid algorithm called Ncorr to correct these erroneous reads. By implementing this Ncorr algorithm [24] they have error corrected the reads of the *E. coli* dataset sequenced by Quick et al. [23] and the reads of the yeast dataset sequenced by Goodwin et al. [24]. These error corrected datasets have been retrieved from the Schatzlab website [24] in FASTA format for all subsequent analysis.

### Composition of the nanopore sequencing datasets used in this study

The *E. coli* dataset consisted of 1,8842 2D reads (when two strands are read accurately and consensus is built from them) as well as 25,432 template and 11,130 complement reads (which cannot be converted to 2D reads) while, the yeast dataset consisted of 28,258 2D reads, 56,046 and 20,506 template and complement reads respectively.

### Assembler algorithms employed in this study

#### Velvet

Velvet (developed in C) is a secure and reliable de Bruijn graph-based assembler. It extensively uses graph simplification strategy to scale down non-intersecting paths into single nodes. This simplification compresses the graph without much loss of information. To reduce the time-complexity of the algorithm, Velvet implements bubble search (to narrow down the candidate bubbles) and read threading (removal of paths that represent fewer reads than the threshold) [29, 31].

#### ABySS

ABySS (developed in C++) is a de Bruijn graph based assembler mainly developed to address the memory issues while assembling mammalian–size genome. ABySS implements a partition approach at the level of individual graph nodes (for efficiency each graph node is processed separately as each node is individually assigned to a CPU). To overcome the memory requirements, the assignment of graph node to CPU is attained by converting K-mer to an integer using strand-neutral formula i.e. k-mer and its reverse complement map to same integer. ABySS also implements graph simplification like Velvet and then performs bubble smoothening by bounded search where priority is given to the path supported by more reads [32].

#### Celera

Celera is an Overlap Layout Consensus (OLC)-based assembler, which was developed at the time of Sanger sequencing by Celera Genomics. In recent years, the algorithm has been modified to handle long Pac Bio reads whose nature is similar to nanopore reads. The revised pipeline is named as CABOG (Celera assembler with best overlap graph). CABOG constructs an overlay graph from the reads and reports the best overlaps, which are then used to build unitigs. These unitigs are joined to build contigs and finally these contigs are connected to form scaffolds [27].

#### SSAKE

SSAKE is a greedy graph-based assembler. It does not use the graph explicitly. Instead, it iteratively searches for reads with overlap to build the contigs. Initially, it will look for reads with end –to –end confirmation by favoring error-free reads and then performs the extension [29].

### Binning of reads

In order to test the performance of various metrics in relation to the size of the datasets, we have divided the total reads in a dataset into four bins i.e. 25 %, 50 %, 75 % and 100 % of the reads. To avoid the prejudice in selecting the reads, the binning of the data was performed by randomly generating the bins of the reads ten times using python script, and finally the average result of all the ten trials after processing the each trial is reported in the figures.

### Implementation of the benchmarking pipeline

According to our survey there are at least 24 de novo assemblers which can be accessed with a free academic license [33], that have been developed by implementing one among the following three algorithms namely de Bruijn graphs, Overlap Layout Consensus (OLC) and greedy extension. Out of these only few assemblers i.e. Velvet [24] (de Bruijn graph), Abyss (de Bruijn graph) [25], Celera (OLC method) [34] and SSAKE (greedy extension based method) [29] could be successfully run for assembling the nanopore reads. All the other assemblers failed to assemble most likely due to the length of the reads and/or due to their expectation for quality scores or other input parameters not available for nanopore reads. It is important to note that most of the assemblers were developed in view of short read sequencing data whose read lengths range from 500-3000 bp [5] whereas the read length of Nanopore reads range from 5000–50,000 bp. All the assemblers in this study were run on UNIX command line with default parameters (to evaluate true potential of each tool), and the obtained results were analyzed for reliability, quality and accuracy of assemblies.

To evaluate and compare the efficiency of various assembly algorithms the following metrics were employed [33]:

#### Calculation of assembly metrics

The contig files which were generated as a result of successful assembly by each assembler were used for statistical analysis of an assembly. The assembly metrics i.e. N50 value,which represents 50 % content of the assembly and all the contig metrics including the mean, total length of all generated contigs as well as the number of contigs obtained, were calculated using a perl script.

#### Calculation of performance metrics

Running times and memory consumed by each assembler was captured during the assembly process using UNIX utilities. While limiting the memory usage of each assembler was accomplished using the Ulimit program.

#### Calculation of accuracy metrics

To assess the accuracy and quality of the generated assemblies, genome coverage i.e. defined as the percentage of the genome covered when the generated contigs are mapped onto reference genome and the percentage of alignment i.e. the number of contigs mapped to the genome out of the total generated contigs, were computed using shell scripts while mapping to the reference genome was performed using a fast gapped aligner tool Bowtie [24].

## Results and discussion

### Pipeline implemented for the analysis

**Fig. 1:**
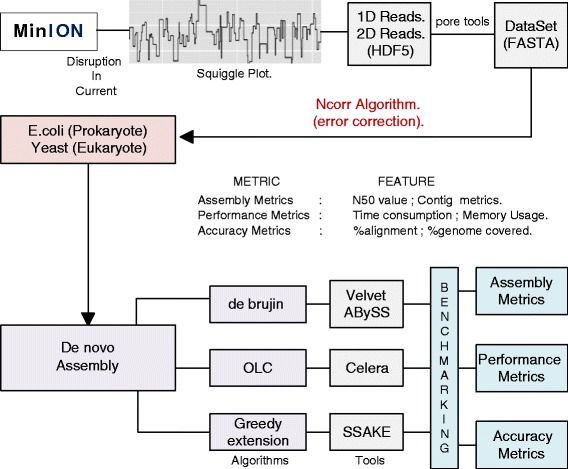
Illustrates the pipeline implemented in this study for benchmarking various assembler algorithms on Nanopore sequenced datasets

Our analysis pipeline shown in Fig. 1, illustrates the step wise protocol followed for benchmarking the various assembler algorithms for Nanopore sequencing data (see Materials and Methods). Initially, we have retrieved the non-error corrected and Ncorr-error corrected [24] datasets to perform preliminary analysis with all the available assemblers, which helped us to identify few assemblers that can potentially assemble Nanopore sequenced reads. After this initial analysis to identify potential assemblers, we analyzed the efficiency of these assemblers as well as the accuracy and quality of the generated assemblies using various metrics on the error-corrected reads. Our benchmarking analysis enabled us to unveil the ideal algorithmic frameworks for addressing the various needs in the assembly of Nanopore sequencing data.

### Comparison of the assembly metrics generated by various assemblers reveals Celera as an optimal assembler

The main features that can best explain the quality of an assembly from sequencing reads include the N50 value, number of contigs, mean length of contigs and the total sum of the lengths of all the contigs identified in an assembly. Hence, we have calculated all of these metrics for the Nanopore sequencing reads for the *E. coli* and Yeast genomes to understand the relative performance of the assemblers (see Materials and Methods). In the following sections, we summarize these comparisons.

**Fig. 2:**
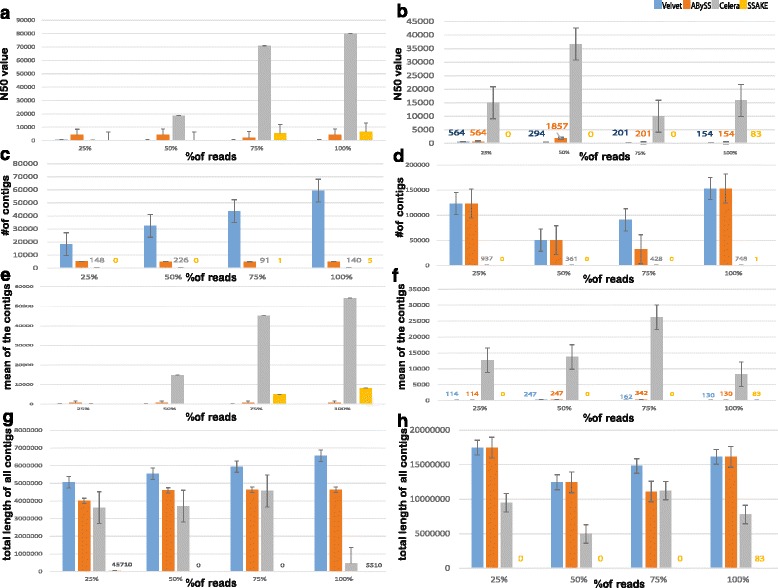
Each pair of plots give an overview of the comparisons of the quality of the assemblies across assemblers for *E. coli* and yeast datasets. **a&b**: Histograms with error bars plotted between % of 2D reads and N50_value of an assembly show the variation in N50 value of an assembly among different assembler algorithms and how it varies with respect to the data size. **c&d**: Histograms with error bars plotted between % of 2D reads and number of contigs generated from an assembly, shows how the number of contigs generated vary with respect to the mean contig length for each respective assembler algorithm across various bins of respective datasets. **e&f**: Histograms showing the percentage of 2D reads employed on X-axis versus the average length of the contigs obtained using each algorithm. **g&h**: Histograms showing the sum of the lengths of all the contigs generated by an assembler as a function of the percentage of the total reads employed in the assembly. In each set of plots, left panel corresponds to *E. coli* dataset while the plots in the right panel correspond to the Yeast dataset. In all the plots labeled numeric values on histograms indicate corresponding values of the metric in respective color representing each tool

1) N50 value: Upon analyzing the 2D reads from *E.coli* dataset (see Fig. 2a) we observed consistent increase in the N50 values of the assemblies generated by various assemblers with an increase in the dataset size measured as the percentage of the total number of reads employed in the analysis. In particular, assembly generated by Celera had the highest N50 value ranging from 20,000 bp to 80,000 bp (as we move from 25 % to 100 % of the total reads) while, the assemblies generated by Velvet, ABySS and SSAKE consisted of an average N50 value of 10,000 bp (approximately), which is eight times lower than N50 value of Celera generated assembly for *E. coli* (Fig. 2a). The differences were found to be even more striking when the results were compared between the assemblers for the yeast genome (Fig. 2b). Since, N50 value represents the 50 % content of the assembly, higher the N50 value better would be the quality of an assembly. Hence, from the above observations based on N50 values, it can be concluded that Celera assembler is likely to generate better assembly compared to the other assemblers studied here. It is possible to speculate that since OLC-based algorithms like the Celera assembler have traditionally been used for longer read technologies like sanger sequencing and probably due to recent modifications made to this specific implementation to make it compatible with even longer reads like PacBio reads of length 3000-15000 bp, they are likely to outperform in terms of assembly quality, most short-read assembler implementations for nanopore sequencing data. This is especially likely to be true if the number of allowed mis-matches for building the contigs can be increased - due to high error rates in the ends of the nanopore reads. While, SSAKE generated assembly is of poor quality with an N50 value of approximately 100 for the yeast genome, which is 100 times less than N50 value of Celera generated assembly (Fig. 2b). A similar trend was observed when template (see Additional file 1) and complement reads (see Additional file 2) of the *E. coli* dataset were analyzed, confirming the reproducibility of the results. Notably, when 2D reads of Yeast dataset were analyzed, we observed that the N50 value of an assembly generated by Celera for 50 % of the reads is much higher than for the whole dataset. Even though the binning of the reads was performed by randomly generating the bins of reads ten times, it is possible to associate this variation due to selection bias or genome-specific variations as this trend was seen only for yeast and not *E. coli*. However, it is still evident that the N50 value of Celera generated assembly is much higher than the N50 values of the assemblies generated by any other assembler (see Fig. 2b). We found very similar trends for 1D reads namely template and complement reads, further confirming the reproducibility of the results (see Additional files 1 and 2).

2) Number of contigs: We observed, that the number of contigs and mean contig length of an assembly are inversely proportional (Fig. 2c–f). Ideally, a good assembler should generate less number of contigs with a high mean and N50 values. We found this generally held true for assemblies generated by Celera compared to the other assemblers studied here. For instance, Velvet followed by ABySS were found to consistently show high number of contigs compared to other assemblers at different percentage of reads employed in the assembly. This was in contrast to the assemblies generated from Celera and SSAKE, which were found to show low number of contigs indicating the possibility of low but more comprehensive assemblies from the latter two (Fig. 2e and f). These results suggest that Velvet and ABySS are likely to produce very fragmented assemblies. We found similar trends for all the three types of reads in both the datasets (see Fig. 2(c, d), Additional files 1 and 2). Since Celera assembler initially constructs overlay graph among reads and reports the best overlaps, which are further used to build untigs, which are joined to generate contigs, it is possible that data from error-corrected long read sequencing technologies like nanopore are likely better assembled using OLC-based methods as the read error-correction methods further improve. Indeed, less number of contigs with longer lengths identified in our analysis by Celera’s assembler further supports this trend.

3) Mean length of the contigs: It is similar to N50 value but the weightage is not given to contigs with longer length while calculating the mean. We found that it followed similar trend in both the *E. coli* and yeast datasets i.e. Celera assembler generating contigs with high mean values followed by Velvet, ABySS and SSAKE respectively (see Fig. 2e and f)). The analysis of 1D reads revealed the same overall trend but the increasing trend of mean values were not found to be proportional with data size unlike that seen for 2D read data (see Additional files 1 and 2).

4) Total sum of lengths of all contigs: While this metric does not play a specific role in assessing the quality of an assembly mainly when the genomes have several duplicated regions, nevertheless it can provide information which can be useful for downstream analysis and prioritization in the assembly framework. So we compared the total length of the contigs obtained, at varying percentages of sequence data employed, using various assemblers (Fig. 2g and h). Not surprisingly, this analysis revealed that the assemblers which showed high number of contigs also exhibited a high total contig length suggesting that these assemblers are likely to produce too many fragmented and/or repetitive contigs thereby causing erroneous assemblies.

Upon analyzing the assembly metrics of the generated assemblies we observe that, irrespective of the data size and its complexity across genomes, OLC based Celera assembler generates better quality assembly than other assemblers.

### Evaluation of the memory and run time requirements of various assemblers reveals Celera to be the fastest when sufficient memory is provided

**Fig. 3:**
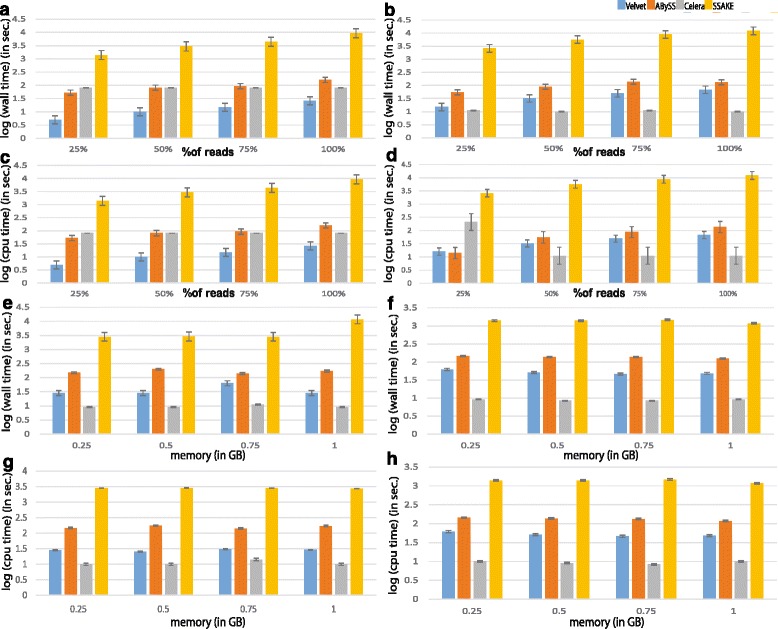
Each pair of plots give an overview of the computational requirements of each assembler for assembling *E. coli* and Yeast datasets. **a&b**: Histogram with error bars plotted between % of 2D reads and log values of wall time which represents the actual time consumed by each assembler to execute the task with respect to gradual increase in data size. **c&d**: Histograms with error bars plotted between % of 2D reads and log values of CPU time which represents amount of time the CPU is actually executing instructions for each assembler with variation in data size. **e&f**: Histograms with error bars plotted between varying amount of allotted memory on X-axis and log values of the wall time, showing the influence of memory allocation on wall time consumption by various assembler algorithms. **g&h**: Histograms with error bars plotted between varying amount of memory and log values of the CPU time, illustrating the influence of memory allocation on the CPU time consumed by various assembler algorithms. In each set of these plots, left panel corresponds to *E. coli* dataset while the plots in the right panel correspond to the Yeast dataset

Major parameters that can be measured to assess the performance of any computational tool or algorithm are memory (virtual and RAM) and time consumed by the tool to complete the assigned task. In this study, we observed that irrespective of size of the dataset, the RAM and virtual memory required for each tool to perform the task is ~26.5 KB and ~1.2 KB respectively. While the time required by each tool to complete the task significantly varies with the size of the dataset and complexity of the genome. For 2D reads of the *E. coli* dataset, the wall time as well the CPU time consumed by Velvet is the lowest with ~15–30 sec of wall time and 15–30 sec of CPU time followed by Celera with ~90 sec each of wall time and CPU time, ABySS with ~50–100 sec of wall time and ~60–100 sec of CPU time and SSAKE with ~1000–1500 sec of wall time and ~1500 sec of CPU time (see Fig. 3a and c, Additional file 3). Values for run times are log transformed in the plots to facilitate easy comparison across tools and datasets. Across the assemblers, the time taken to run by each tool increased with the increase in the data size. For yeast dataset, the trend was found to be same but the time consumed by each tool was approximately 3 times higher than the time consumed to assemble the *E. coli* genome, likely due to the differences in the complexity of the genomes and size of the datasets (see Fig. 3b and d, Additional file 4). In addition, we analyzed the performance of the assemblers, by restricting the memory allotment using Ulimit utility on UNIX environment, to study how the run times vary across them when memory allotted is altered between different runs. We observed that the time taken by each tool remains same when more amount of memory is provided except for Celera, for which we found that the run times significantly decreased when more memory is provided and this resulted in a trend with Celera consuming the lowest time followed by Velvet, ABySS and SSAKE (see Fig. 3e–h, Additional files 3 and 4). The analysis of 1D reads further confirmed the reproducibility of these results (see Additional files 5 and 6).

Overall, our performance metric analysis revealed that the time taken by the de Bruijn graph and OLC-based algorithms to generate assembly is low, while the time consumed by greedy-extension algorithms to generate the assembly are likely to be relatively higher for nanopore data. This might be due to the extensive search made by the greedy-extension algorithms to find the end-to-end overlap of the reads while assembling. It is possible that indexing in greedy extension methods might reduce the run times to some extent. On other hand, de Bruijin graph based assemblers take less time as they implement bubble search which narrow down the candidate bubbles and help in speeding up the assembly process. While, Celera implements OLC algorithm which looks for overlap among the reads to join them together. Since, nanopore reads are longer but fewer, it is not only easy to find overlaps but are also likely to exhibit longer overlaps among the reads, which facilitates more accurate construction of the Contigs. Thus, it is possible that OLC-based approaches like Celera will take lesser run time to generate more accurate assemblies with nanopore data. However, in order to improve performance of these methods, it is important to note that error rates in nanopore reads need to be decreased while allowing increased mismatches in the assembly process.

### Evaluation of the quality of the generated assemblies reveals OLC-based algorithms to be ideal for nanopore data

**Fig. 4:**
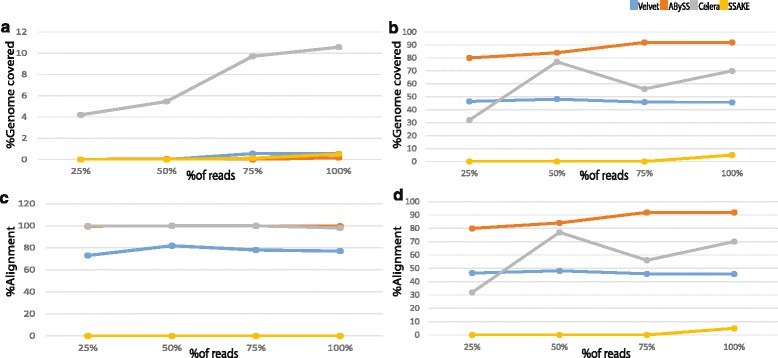
Each pair of plots show the accuracy of the assembly generated by various assembler algorithms for *E.coli* (Panels A and C) and Yeast (Panels B and D) datasets. **a&b**: Line graphs plotted between % of 2D reads and the % of genome covered, showing the extent of genome assembled by each assembler algorithm. **c&d**: Line graphs between the % of 2D reads and % of alignment showing the confidence level of the contigs being assembled by various assembler algorithms

Two specific metrics which can help in assessing the accuracy of an assembly are genome coverage and alignment percentage (see Materials and Methods). Surprisingly, the genome coverage of all the generated assemblies was very low, but comparatively the assembly generated by Celera for the *E. coli* 2D read data exhibited better genome coverage (12–13 % versus 2 % for all other assemblies) (see Fig. 4a). For the yeast dataset, the percentage of genome coverage for the assemblies generated by ABySS, Celera and Velvet were found to be 80 %, 70 % and 50 % respectively. In contrast, it was found to be only 2 % for the assembly generated by SSAKE (see Fig. 4b). When the percentage of alignment was compared between the assemblers, the contigs generated by Celera and ABySS for the *E. coli* 2D read data showed 100 % alignment to the reference genome while the alignment percentage of the contigs generated by Velvet and SSAKE was found to be 80 % and 0 % respectively (see Fig. 4c). For Yeast 2D read data the alignment percentage ranged between 60 %–90 %, with contigs generated by ABySS having highest alignment percentage when aligned to the reference genome followed by Celera, Velvet and SSAKE (see Fig 4d). Further evaluation of 1D reads for coverage and alignment showed a similar trend, confirming the reproducibility of these results (see Additional files 7 and 8).

## Conclusion

In this study, we implemented a computational pipeline for the benchmarking of assembler algorithms which revealed several observations which can aid in the development and improvement of frameworks for assembling genomes using nanopore data. In particular, we found that OLC-based assembler Celera generates an assembly with ten times higher N50 value & mean value and five times lower number of contigs. Our analysis also confirmed that OLC-based approaches can result in high genome coverages with 12 % in *E. coli* and 70 % in Yeast along with moderate alignment percentages of approximately 85 % when compared to other assemblies, indicating a relatively high quality of the assembly compared to other tools studied here. Moreover, Celera was found to exhibit lesser run times when increased memory was provided to perform the task. Thus, Overlap Layout Consensus (OLC) based algorithms would be ideal frameworks for building de novo assemblers for nanopore reads followed by de Bruijn graph based algorithms since assemblies generated by ABySS were found to show high accuracy, moderate quality and reasonable run times and memory requirements. Our results also suggest that improvements in greedy-extension algorithms can be implemented by indexing in order to decrease the run times. Although this step might reduce the run times for greedy extension methods, accuracy and quality of an assembly generated will be potential issues to be addressed for these methods.

There are several challenges that currently exist in dealing with the Nanopore sequencing data. These include high error rate of the long reads and lack of automated computational pipelines for error correction, assembly/alignment as well as downstream analysis of the reads. Developing efficient algorithms which can automate the process of error correction and assembly of the reads would pose some potential opportunities in this domain. For instance, an automated pipeline can be developed by implementing HGAP (Hierarchical Genome Assembly Process) algorithm for error correction, which is already proven to be an optimal algorithm for the error correction in the context to PacBio reads. However, the implementation of HGAP algorithm restricts the application of the tool to specific genomes i.e., only those for which short read data is already available in the public domain. Hence, there is a need to develop methods which can correct the reads from single molecule sequencing methods without using short read or reference genome sequences and using such implementation in the assembly and alignment process for downstream analysis. Indeed, we anticipate rapid development of automated computational pipelines to address various aspects of nanopore sequencing data analysis as new datasets spanning multiple species become available to the scientific community in the coming years. Hence, some of the opportunities for computational biologists include:

1. Enhancing the error correcting algorithms which don’t require short read sequencing data or reference genomes.
2. Development of OLC based assembler algorithms which can consider error-rates in the assembly process, since our results confirm the performance of these methods to be significantly better than other algorithms.
3. Developing automated pipelines for pre- processing of the long reads and downstream analysis.

## Abbreviations

Bp, base pair; kbp - kilobasepair; *E. coli*, *Escherichia coli;* MAP, MinION^®^ early Access Program; *OLC*, overlap layout consensus; Yeast, *Saccharomyces cerevisiae*

## Additional files

Additional file 1: Each pair of plots give an overview of the comparisons of the quality of the assemblies across assemblers for nanopore sequenced template reads from *E. coli* and yeast datasets. A&B: Histograms with error bars plotted between % of template reads and N50_value of an assembly show the variation in N50 value of an assembly among different assembler algorithms and how it varies with respect to the data size. C&D: Histograms with error bars plotted between % of template reads and number of contigs generated from an assembly showing how the number of contigs generated vary for each respective assembler algorithm across various bins of respective datasets. E&F: Histograms showing the percentage of template reads employed on X-axis versus the average length of the contigs represented as mean of the contigs, obtained using each algorithm. Mean of the Contigs is the average value of the total sum of lengths of all the contigs. G&H: Histograms showing the sum of the lengths of all the contigs generated by an assembler as a function of the percentage of the total reads employed in the assembly. In each set of plots, left panel corresponds to *E. coli* dataset while the plots in the right panel correspond to the Yeast dataset. In all the plots labeled numeric values on histograms indicate corresponding values of the metric in respective color representing each tool. (PDF 670 kb) Additional file 2: Each pair of plots give an overview of the comparisons of the quality of the assemblies across assemblers for nanopore sequenced complement reads from *E. coli* and yeast datasets. A&B: Histograms with error bars plotted between % of complement reads and N50_value of an assembly show the variation in N50 value of an assembly among different assembler algorithms and how it varies with respect to the data size. C&D: Histograms with error bars plotted between % of complement reads and number of contigs generated from an assembly showing how the number of contigs generated vary for each respective assembler algorithm across various bins of respective datasets. E&F: Histograms showing the percentage of complement reads employed on X-axis versus the average length of the contigs represented as mean of the contigs, obtained using each algorithm. Mean of the Contigs is the average value of the total sum of lengths of all the contigs. G&H: Histograms showing the sum of the lengths of all the contigs generated by an assembler as a function of the percentage of the total reads employed in the assembly. In each set of plots, left panel corresponds to *E. coli* dataset while the plots in the right panel correspond to the Yeast dataset. In all the plots labeled numeric values on histograms indicate corresponding values of the metric in respective color representing each tool. (PDF 700 kb) Additional file 3: Overview of the running times for various assemblers. (PDF 236 kb) Additional file 4: Overview of the influence of memory allocation on the running times for various assemblers. (PDF 155 kb) Additional file 5: Each pair of plots give an overview of the computational requirements of each assembler for assembling nanopore sequenced template reads from *E. coli* and Yeast datasets. A&B: Histogram with error bars plotted between % of template reads and log values of wall time which represents the actual time consumed by each assembler to execute the task with respect to gradual increase in data size. C&D: Histograms with error bars plotted between % of template reads and log values of CPU time which represents amount of time the CPU is actually executing instructions for each assembler with variation in data size. E&F: Histograms with error bars plotted between varying amount of allotted memory on X-axis and log values of the wall time, showing the influence of memory allocation on wall time consumption by various assembler algorithms. G&H: Histograms with error bars plotted between varying amount of memory and log values of the CPU time, illustrating the influence of memory allocation on the CPU time consumed by various assembler algorithms. In each set of these plots, left panel corresponds to *E. coli* dataset while the plots in the right panel correspond to the Yeast dataset. (PDF 813 kb) Additional file 6: Each pair of plots give an overview of the computational requirements of each assembler for assembling nanopore sequenced complement reads from *E. coli* and Yeast datasets. A&B: Histogram with error bars plotted between % of complement reads and log values of wall time which represents the actual time consumed by each assembler to execute the task with respect to gradual increase in data size. C&D: Histograms with error bars plotted between % of complement reads and log values of CPU time which represents amount of time the CPU is actually executing instructions for each assembler with variation in data size. E&F: Histograms with error bars plotted between varying amount of allotted memory on X-axis and log values of the wall time, showing the influence of memory allocation on wall time consumption by various assembler algorithms. G&H: Histograms with error bars plotted between varying amount of memory and log values of the CPU time, illustrating the influence of memory allocation on the CPU time consumed by various assembler algorithms. In each set of these plots, left panel corresponds to *E. coli* dataset while the plots in the right panel correspond to the Yeast dataset. (PDF 838 kb) Additional file 7: Each pair of plots show the accuracy of the assembly generated by various assembler algorithms for nanopore sequenced template reads from *E.coli* (Panels A and C) and Yeast (Panels B and D) datasets. A&B: Line graphs plotted between % of template reads and the % of genome covered, showing the extent of genome assembled by each assembler algorithm. C&D: Line graphs between the % of template reads and % of alignment showing the confidence level of the contigs being assembled by various assembler algorithms. (PDF 584 kb) Additional file 8 Each pair of plots show the accuracy of the assembly generated by various assembler algorithms for nanopore sequenced complement reads from *E.coli* (Panels A and C) and Yeast (Panels B and D) datasets. A&B: Line graphs plotted between % of complement reads and the % of genome covered, showing the extent of genome assembled by each assembler algorithm. C&D: Line graphs between the % of complement reads and % of alignment showing the confidence level of the contigs being assembled by various assembler algorithms. (PDF 798 kb)
