# Supplementary figures and images for: Benchmarking of de novo assembly algorithms for Nanopore data reveals optimal performance of OLC approaches
Source: BMC Genomics. 2016 Aug 22;17(Suppl 7):507. doi: 10.1186/s12864-016-2895-8 (PMC5001211; doi:10.1186/s12864-016-2895-8)

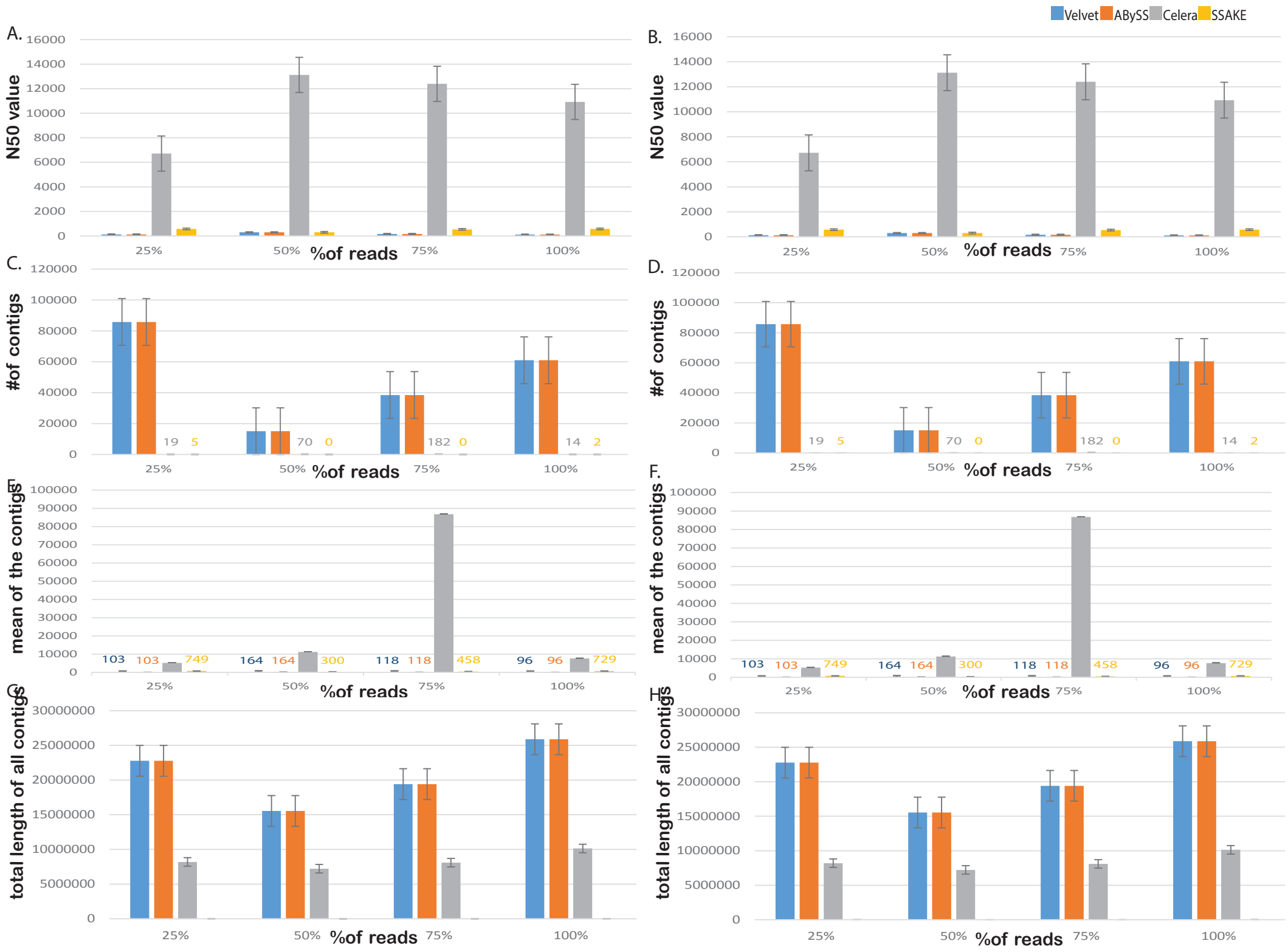

Supplement: Additional file 1: — Each pair of plots give an overview of the comparisons of the quality of the assemblies across assemblers for nanopore sequenced template reads from E. coli and yeast datasets. A&B: Histograms with error bars plotted between % of template reads and N50_value of an assembly show the variation in N50 value of an assembly among different assembler algorithms and how it varies with respect to the data size. C&D: Histograms with error bars plotted between % of template reads and number of contigs generated from an assembly showing how the number of contigs generated vary for each respective assembler algorithm across various bins of respective datasets. E&F: Histograms showing the percentage of template reads employed on X-axis versus the average length of the contigs represented as mean of the contigs, obtained using each algorithm. Mean of the Contigs is the average value of the total sum of lengths of all the contigs. G&H: Histograms showing the sum of the lengths of all the contigs generated by an assembler as a function of the percentage of the total reads employed in the assembly. In each set of plots, left panel corresponds to E. coli dataset while the plots in the right panel correspond to the Yeast dataset. In all the plots labeled numeric values on histograms indicate corresponding values of the metric in respective color representing each tool. (PDF 670 kb) [file 12864_2016_2895_MOESM1_ESM.pdf]

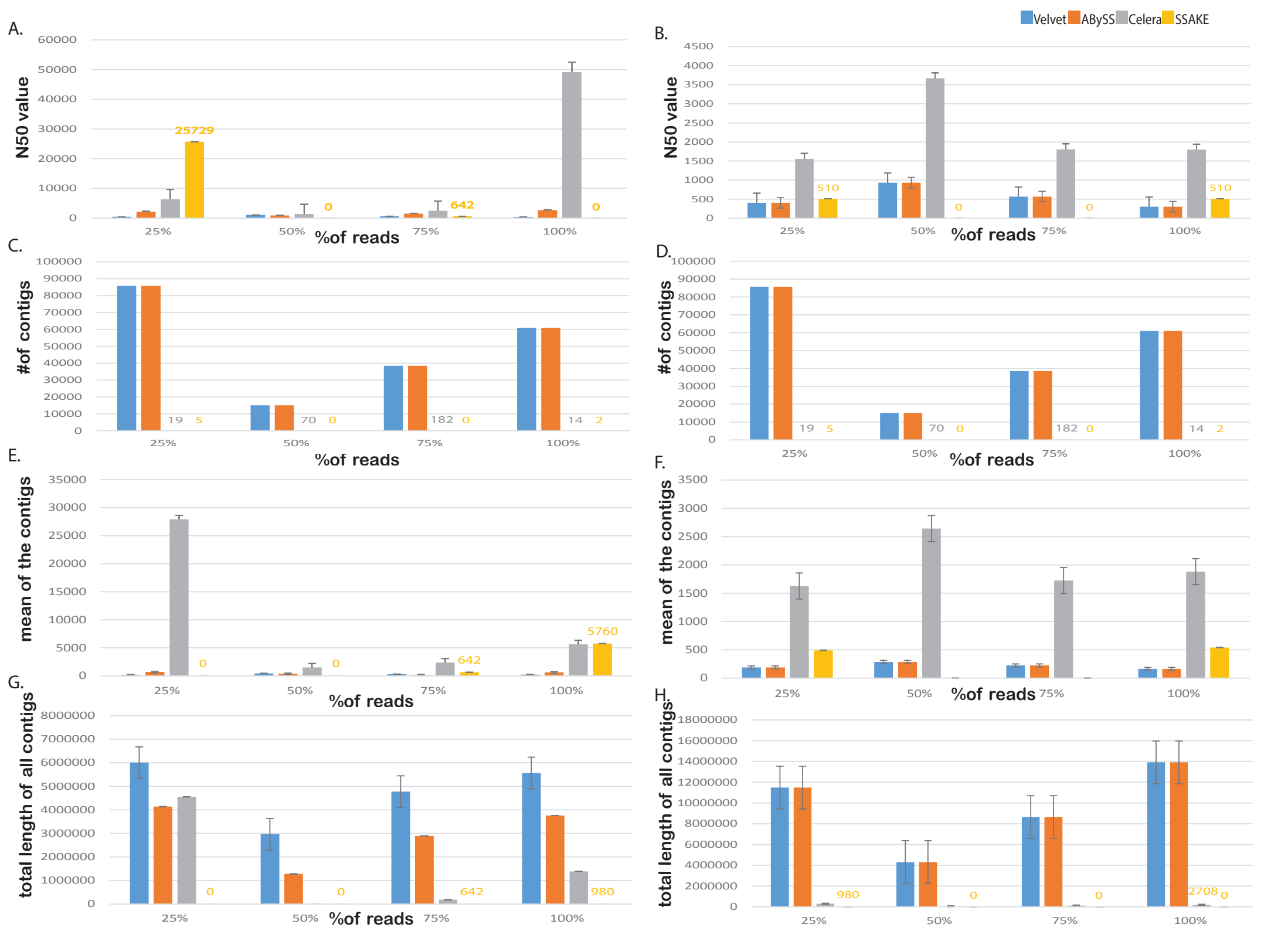

Supplement: Additional file 2: — Each pair of plots give an overview of the comparisons of the quality of the assemblies across assemblers for nanopore sequenced complement reads from E. coli and yeast datasets. A&B: Histograms with error bars plotted between % of complement reads and N50_value of an assembly show the variation in N50 value of an assembly among different assembler algorithms and how it varies with respect to the data size. C&D: Histograms with error bars plotted between % of complement reads and number of contigs generated from an assembly showing how the number of contigs generated vary for each respective assembler algorithm across various bins of respective datasets. E&F: Histograms showing the percentage of complement reads employed on X-axis versus the average length of the contigs represented as mean of the contigs, obtained using each algorithm. Mean of the Contigs is the average value of the total sum of lengths of all the contigs. G&H: Histograms showing the sum of the lengths of all the contigs generated by an assembler as a function of the percentage of the total reads employed in the assembly. In each set of plots, left panel corresponds to E. coli dataset while the plots in the right panel correspond to the Yeast dataset. In all the plots labeled numeric values on histograms indicate corresponding values of the metric in respective color representing each tool. (PDF 700 kb) [file 12864_2016_2895_MOESM2_ESM.pdf]

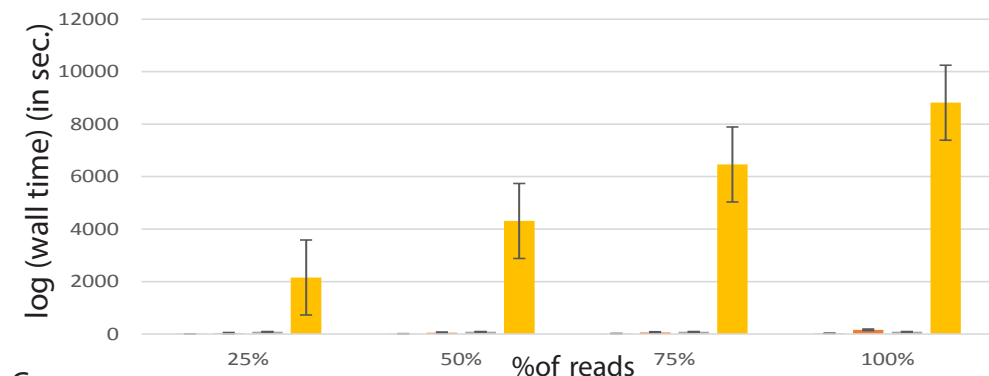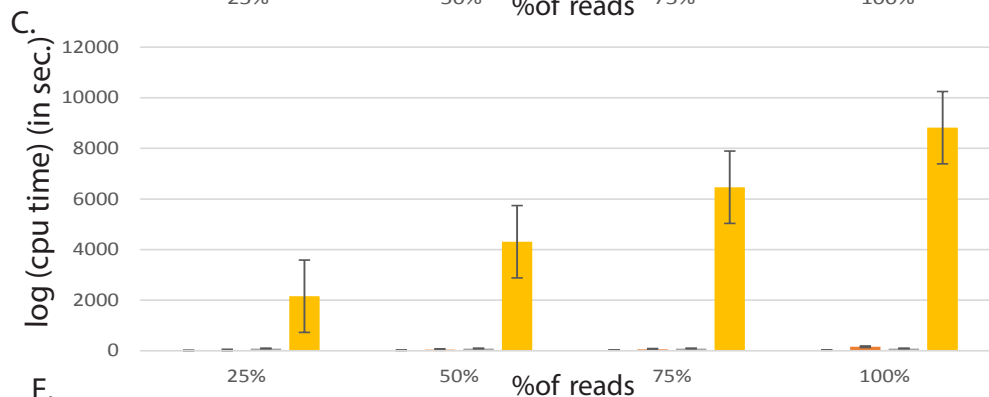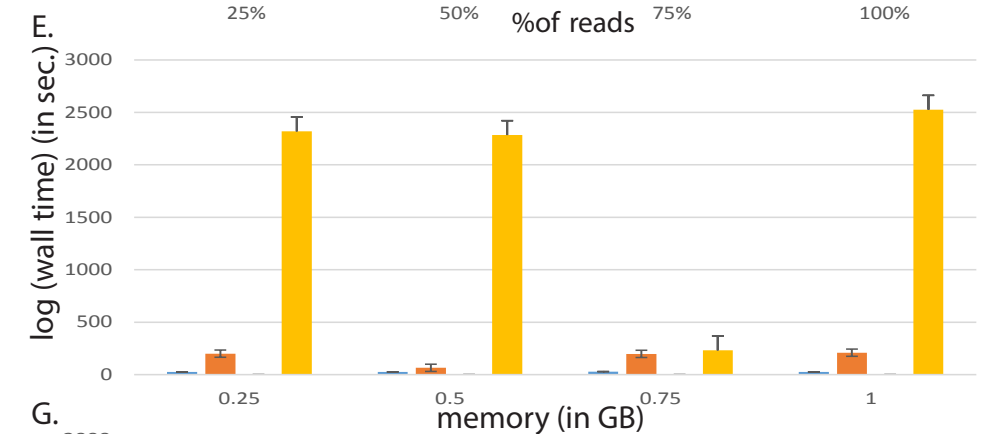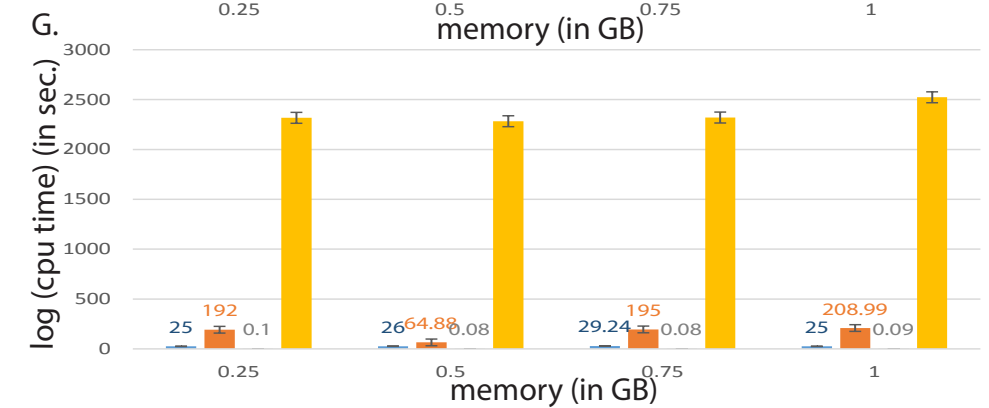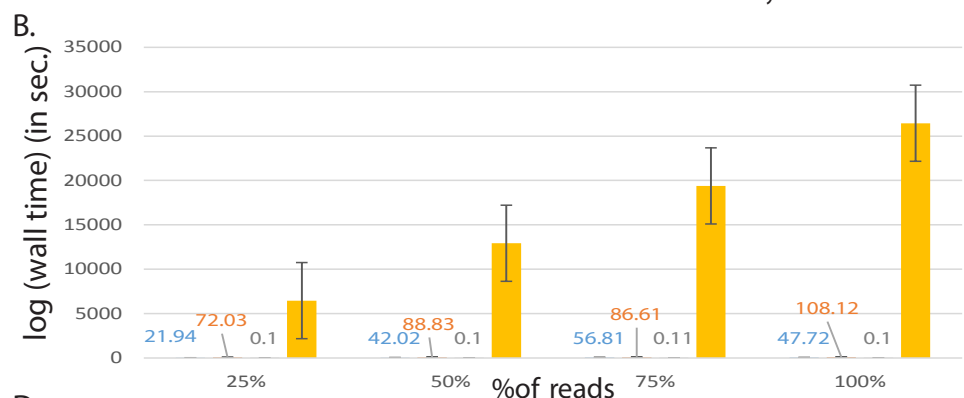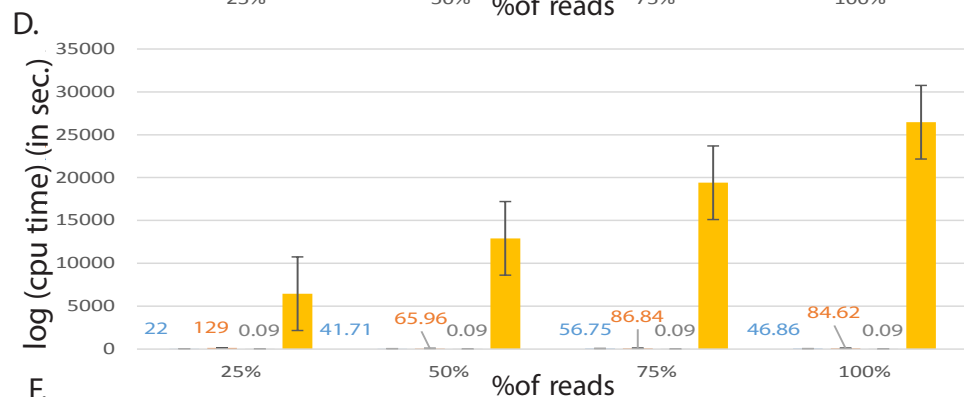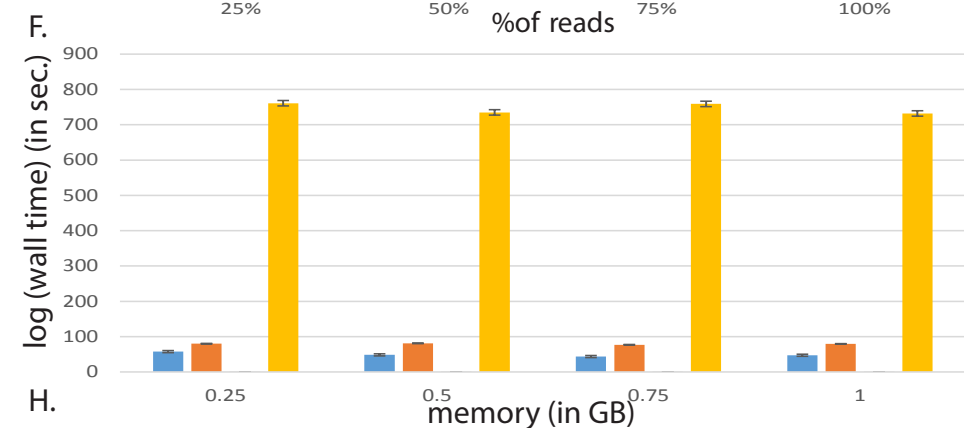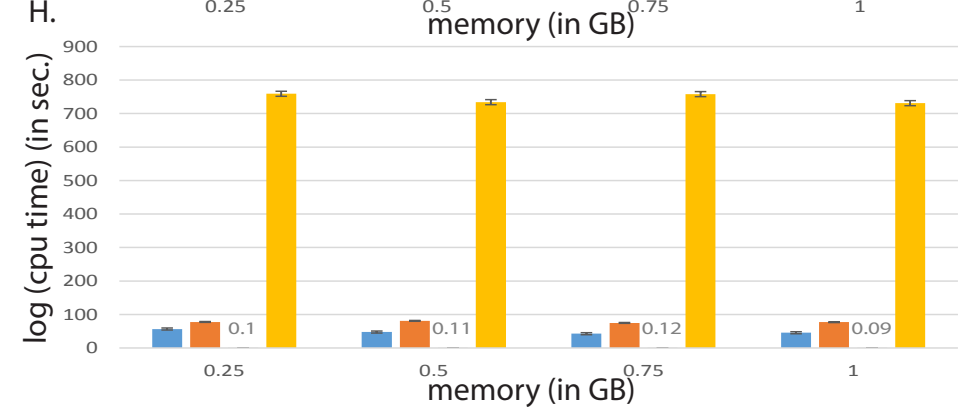

Supplement: Additional file 5: — Each pair of plots give an overview of the computational requirements of each assembler for assembling nanopore sequenced template reads from E. coli and Yeast datasets. A&B: Histogram with error bars plotted between % of template reads and log values of wall time which represents the actual time consumed by each assembler to execute the task with respect to gradual increase in data size. C&D: Histograms with error bars plotted between % of template reads and log values of CPU time which represents amount of time the CPU is actually executing instructions for each assembler with variation in data size. E&F: Histograms with error bars plotted between varying amount of allotted memory on X-axis and log values of the wall time, showing the influence of memory allocation on wall time consumption by various assembler algorithms. G&H: Histograms with error bars plotted between varying amount of memory and log values of the CPU time, illustrating the influence of memory allocation on the CPU time consumed by various assembler algorithms. In each set of these plots, left panel corresponds to E. coli dataset while the plots in the right panel correspond to the Yeast dataset. (PDF 813 kb) [file 12864_2016_2895_MOESM5_ESM.pdf]

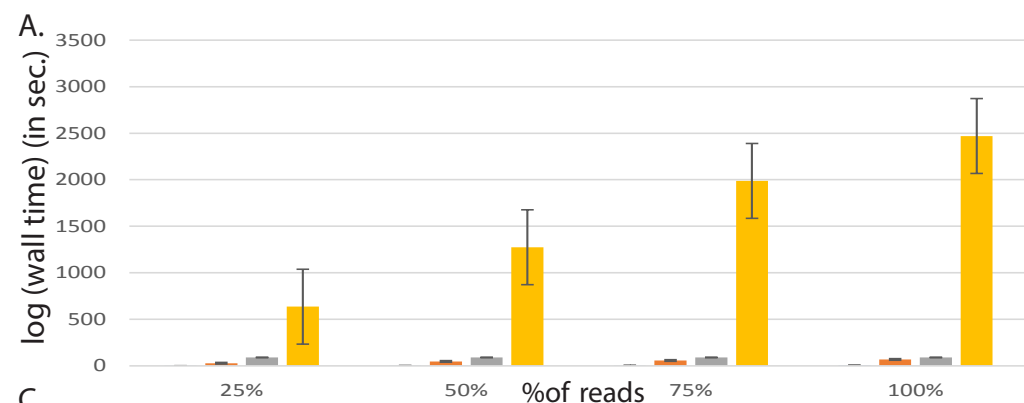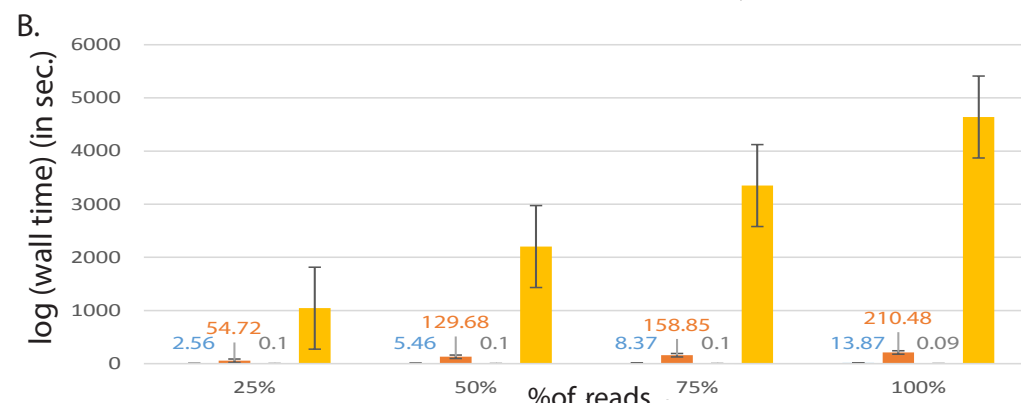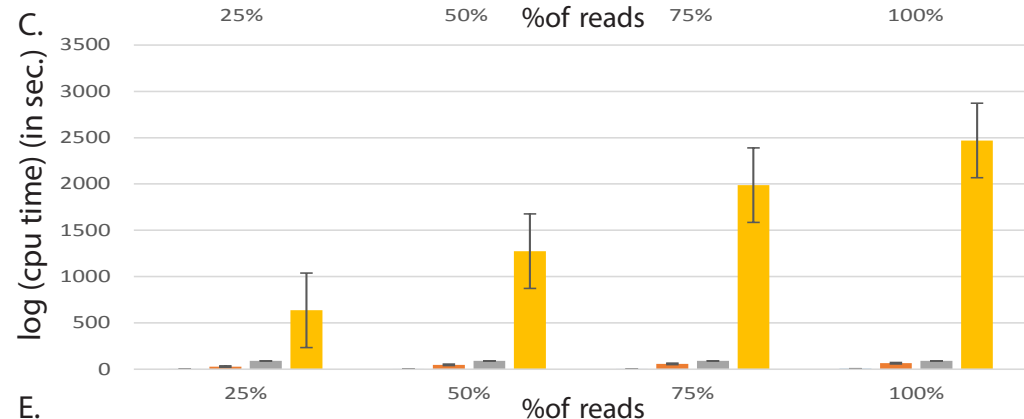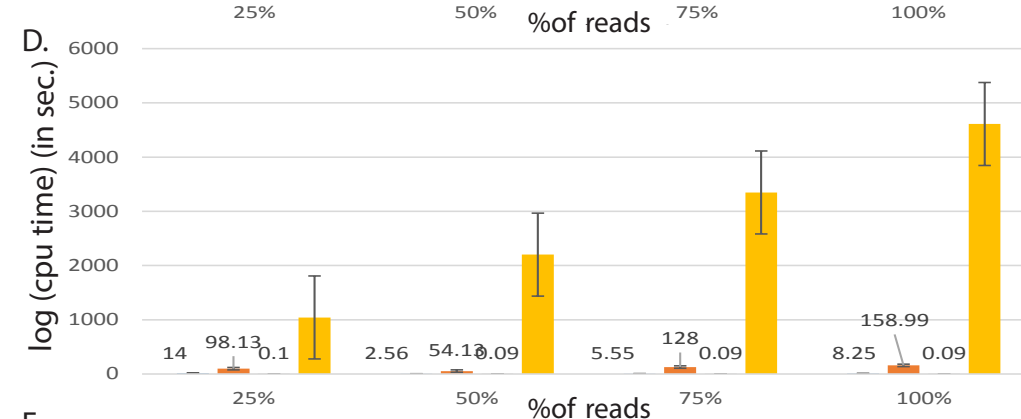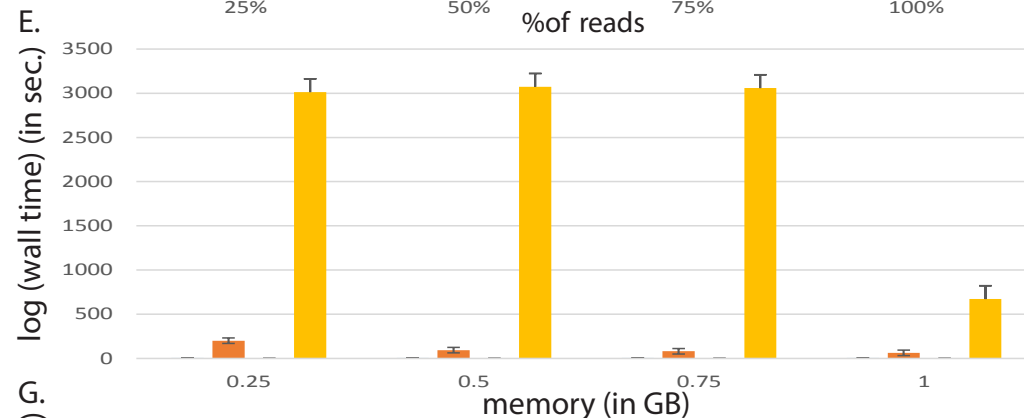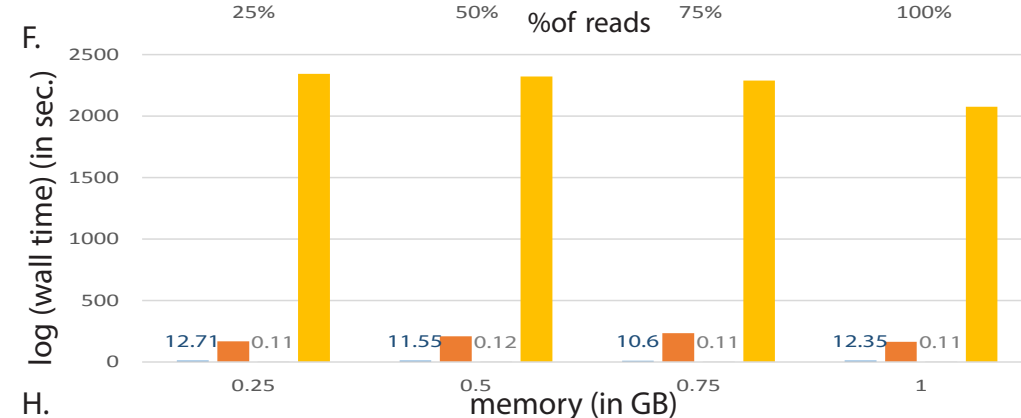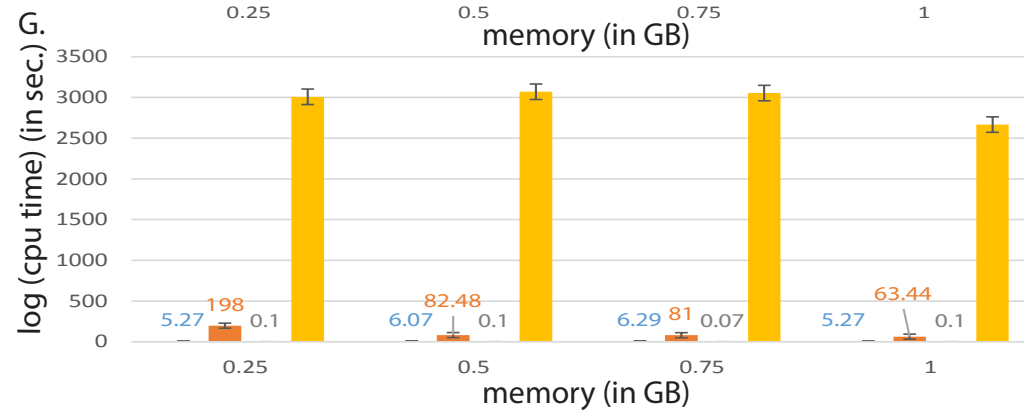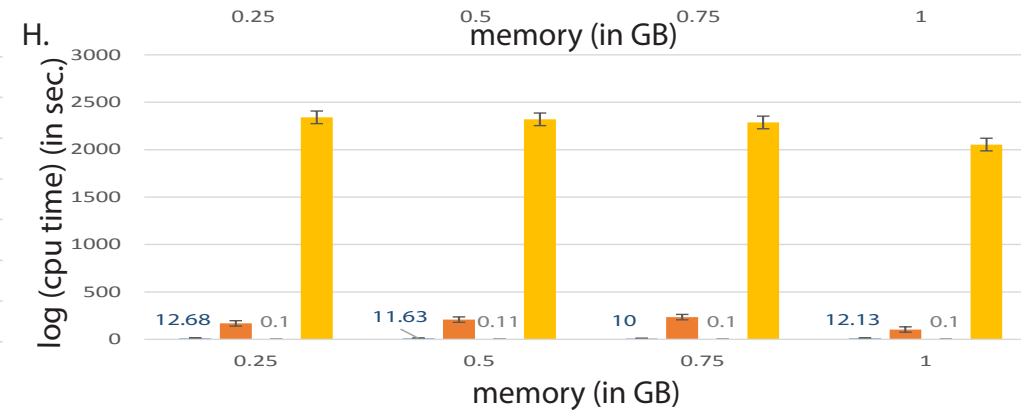

Supplement: Additional file 6: — Each pair of plots give an overview of the computational requirements of each assembler for assembling nanopore sequenced complement reads from E. coli and Yeast datasets. A&B: Histogram with error bars plotted between % of complement reads and log values of wall time which represents the actual time consumed by each assembler to execute the task with respect to gradual increase in data size. C&D: Histograms with error bars plotted between % of complement reads and log values of CPU time which represents amount of time the CPU is actually executing instructions for each assembler with variation in data size. E&F: Histograms with error bars plotted between varying amount of allotted memory on X-axis and log values of the wall time, showing the influence of memory allocation on wall time consumption by various assembler algorithms. G&H: Histograms with error bars plotted between varying amount of memory and log values of the CPU time, illustrating the influence of memory allocation on the CPU time consumed by various assembler algorithms. In each set of these plots, left panel corresponds to E. coli dataset while the plots in the right panel correspond to the Yeast dataset. (PDF 838 kb) [file 12864_2016_2895_MOESM6_ESM.pdf]

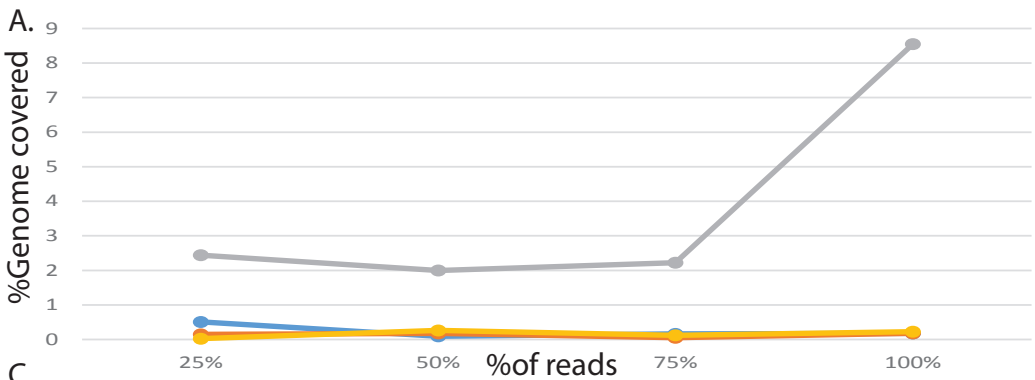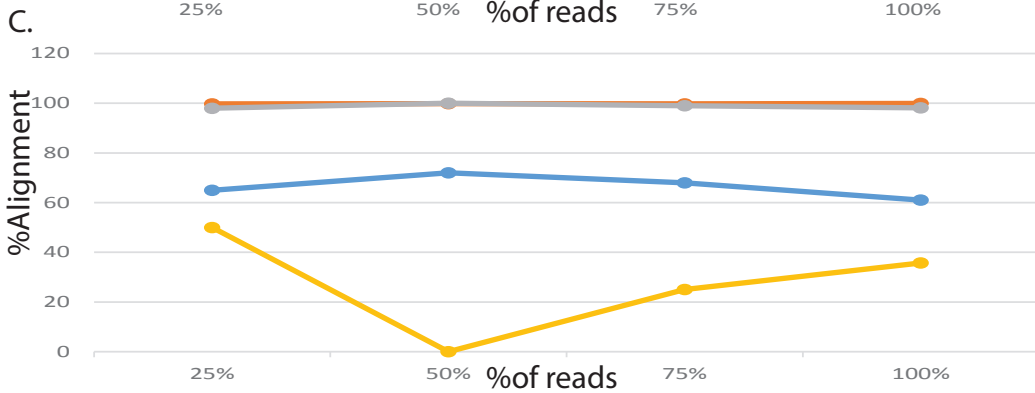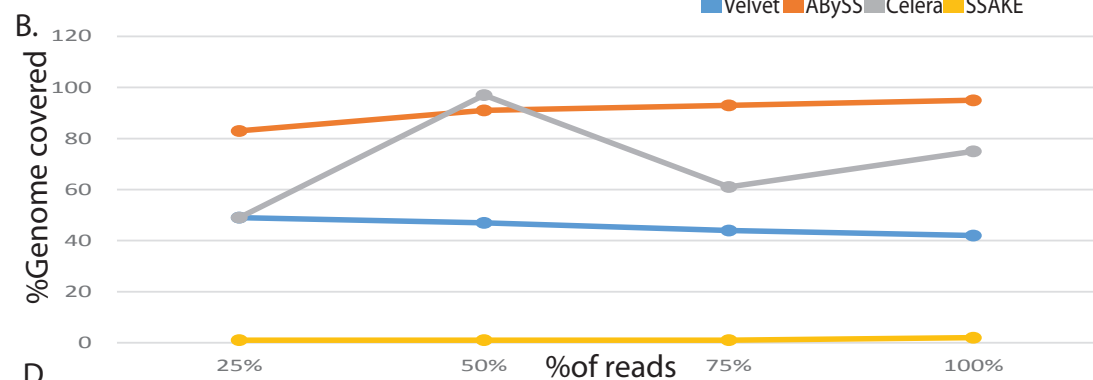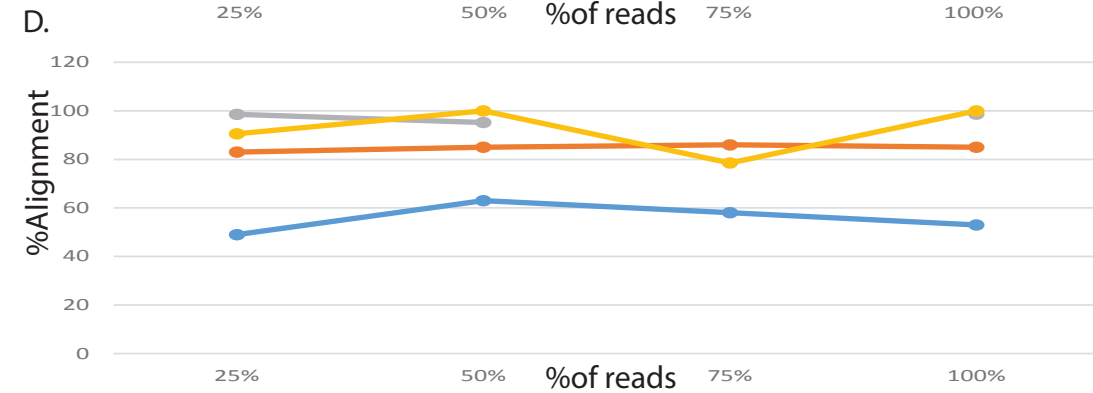

Supplement: Additional file 7: — Each pair of plots show the accuracy of the assembly generated by various assembler algorithms for nanopore sequenced template reads from E.coli (Panels A and C) and Yeast (Panels B and D) datasets. A&B: Line graphs plotted between % of template reads and the % of genome covered, showing the extent of genome assembled by each assembler algorithm. C&D: Line graphs between the % of template reads and % of alignment showing the confidence level of the contigs being assembled by various assembler algorithms. (PDF 584 kb) [file 12864_2016_2895_MOESM7_ESM.pdf]

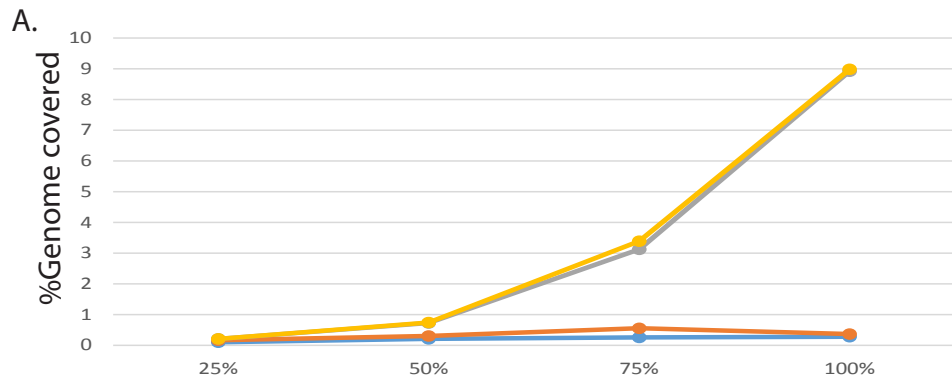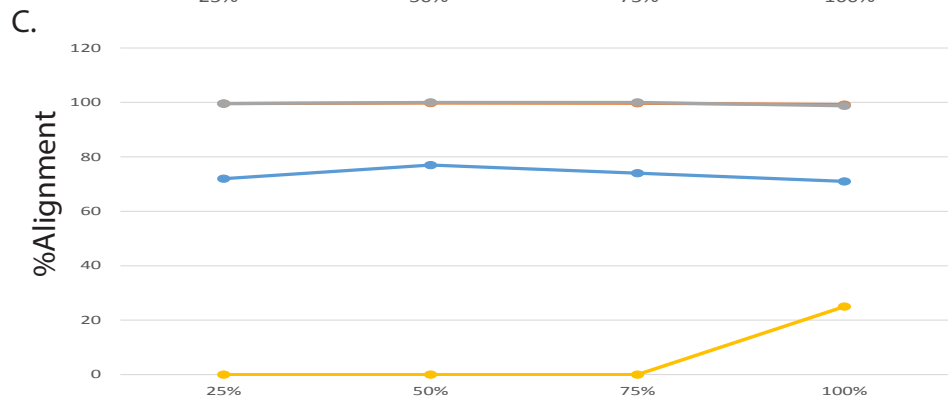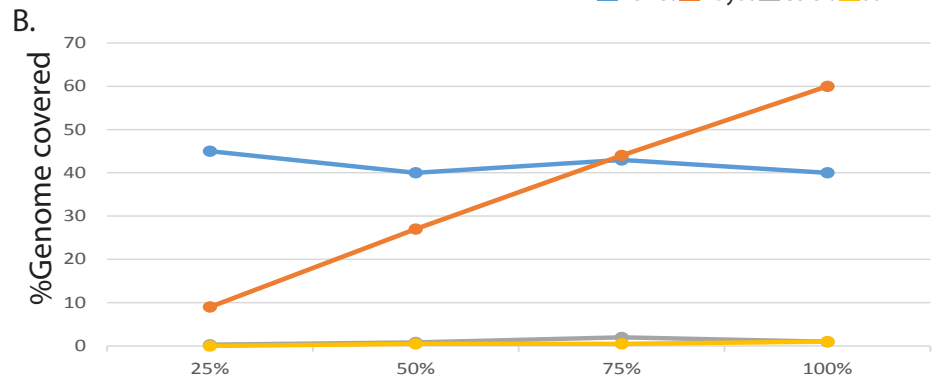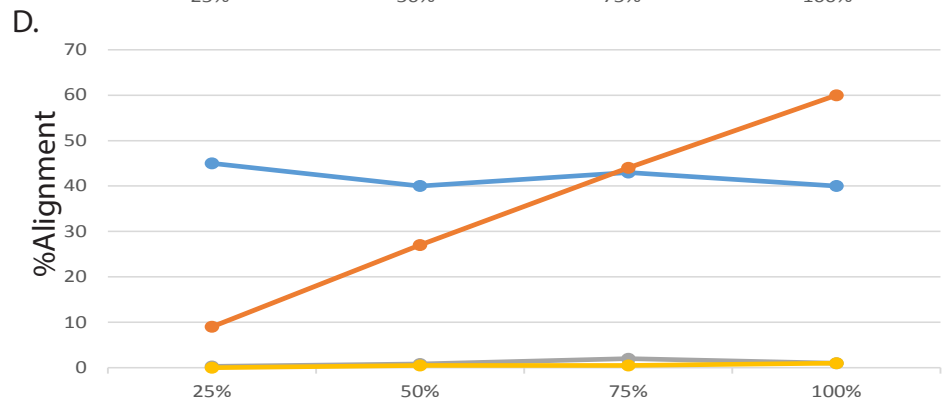

Supplement: Additional file 8 — Each pair of plots show the accuracy of the assembly generated by various assembler algorithms for nanopore sequenced complement reads from E.coli (Panels A and C) and Yeast (Panels B and D) datasets. A&B: Line graphs plotted between % of complement reads and the % of genome covered, showing the extent of genome assembled by each assembler algorithm. C&D: Line graphs between the % of complement reads and % of alignment showing the confidence level of the contigs being assembled by various assembler algorithms. (PDF 798 kb) [file 12864_2016_2895_MOESM8_ESM.pdf]
