# Supplementary material for: Benchmarking of de novo assembly algorithms for Nanopore data reveals optimal performance of OLC approaches
Source: BMC Genomics. 2016 Aug 22;17(Suppl 7):507. doi: 10.1186/s12864-016-2895-8 (PMC5001211; doi:10.1186/s12864-016-2895-8)
Supplement: Additional file 3: — Overview of the running times for various assemblers. (PDF 236 kb) [file 12864_2016_2895_MOESM3_ESM.pdf]

**Additional File 3:** Overview of the running times for various assemblers

| Assembler | Reads type | % of reads | Dataset | wall time (in sec.) | log(wall time) | cpu time(in sec.) | log(cpu time) |
|---|---|---|---|---|---|---|---|
| Velvet | 2D | 25 | E.coli | 5.54 | 0.75 | 5.53 | 1.05 |
| Velvet | 2D | 50 | E.coli | 11.05 | 1.05 | 11 | 1.22 |
| Velvet | 2D | 75 | E.coli | 16.56 | 1.22 | 16.54 | 1.47 |
| Velvet | 2D | 100 | E.coli | 28.42 | 1.46 | 29 | 1.77 |
| Abyss | 2D | 25 | E.coli | 58.29 | 1.77 | 58.4 | 1.96 |
| Abyss | 2D | 50 | E.coli | 123.4 | 2.1 | 90 | 2.02 |
| Abyss | 2D | 75 | E.coli | 103.47 | 2.02 | 103 | 2.26 |
| Abyss | 2D | 100 | E.coli | 34152 | 4.54 | 178 | 1.96 |
| celera | 2D | 25 | E.coli | 90.11 | 1.96 | 90.1 | 1.96 |
| celera | 2D | 50 | E.coli | 90.11 | 1.96 | 90.1 | 1.96 |
| celera | 2D | 75 | E.coli | 90.11 | 1.96 | 90.1 | 1.96 |
| celera | 2D | 100 | E.coli | 90.11 | 1.96 | 90.1 | 3.19 |
| SSAKE | 2D | 25 | E.coli | 1548 | 3.19 | 1545 | 3.52 |
| SSAKE | 2D | 50 | E.coli | 3275 | 3.52 | 3273 | 3.69 |
| SSAKE | 2D | 75 | E.coli | 4900 | 3.7 | 4896 | 4.01 |
| SSAKE | 2D | 100 | E.coli | 39894 | 4.61 | 10228 | 1.21 |
| Velvet | 2D | 25 | yeast | 15 | 1.18 | 16 | 1.52 |
| Velvet | 2D | 50 | yeast | 32 | 1.51 | 32.37 | 1.7 |
| Velvet | 2D | 75 | yeast | 50 | 1.7 | 49 | 1.84 |
| Velvet | 2D | 100 | yeast | 67.99 | 1.84 | 67.8 | 1.15 |
| Abyss | 2D | 25 | yeast | 54.74 | 1.74 | 14 | 1.74 |
| Abyss | 2D | 50 | yeast | 88.52 | 1.95 | 54.74 | 1.94 |
| Abyss | 2D | 75 | yeast | 138.77 | 2.15 | 86.52 | 2.14 |
| Abyss | 2D | 100 | yeast | 131.75 | 2.12 | 135.74 | 2.33 |
| celera | 2D | 25 | yeast | 0.09 | 1.05 | 210.48 | 1.05 |
| celera | 2D | 50 | yeast | 0.1 | 1 | 0.09 | 0.05 |
| celera | 2D | 75 | yeast | 0.09 | 1.05 | 0.9 | 1.05 |
| celera | 2D | 100 | yeast | 0.1 | 1 | 0.09 | 3.42 |
| SSAKE | 2D | 25 | yeast | 2609 | 3.42 | 2607.39 | 3.76 |
| SSAKE | 2D | 50 | yeast | 5648.78 | 3.76 | 5643.65 | 3.95 |
| SSAKE | 2D | 75 | yeast | 8837.37 | 3.95 | 8829.93 | 4.09 |
| SSAKE | 2D | 100 | yeast | 12174.24 | 4.09 | 12165.74 | 0.75 |
