# Supplementary material for: Benchmarking of de novo assembly algorithms for Nanopore data reveals optimal performance of OLC approaches
Source: BMC Genomics. 2016 Aug 22;17(Suppl 7):507. doi: 10.1186/s12864-016-2895-8 (PMC5001211; doi:10.1186/s12864-016-2895-8)
Supplement: Additional file 4: — Overview of the influence of memory allocation on the running times for various assemblers. (PDF 155 kb) [file 12864_2016_2895_MOESM4_ESM.pdf]

**Additional File 4:** Overview of the influence of memory allocation on the running times for various assemblers.

| Dataset | Assembler | Reads type (fulldata) | virtual memory (in GB) | wall time. (in sec.) | log(wall time) | CPU time. (in sec.) | log(cpu time) |
|---|---|---|---|---|---|---|---|
| Ecoli. | Velvet | 2D | 0.25 | 28.23 | 1.46 | 28 | 1.45 |
| Ecoli. | Velvet | 2D | 0.5 | 28.4 | 1.46 | 25.8 | 1.42 |
| Ecoli. | Velvet | 2D | 0.75 | 63.06 | 1.8 | 31 | 1.5 |
| Ecoli. | Velvet | 2D | 1 | 28.23 | 1.46 | 29 | 1.47 |
| Ecoli. | Abyss | 2D | 0.25 | 148.77 | 2.18 | 148 | 2.18 |
| Ecoli. | Abyss | 2D | 0.5 | 198.3 | 2.3 | 176 | 2.25 |
| Ecoli. | Abyss | 2D | 0.75 | 140.86 | 2.15 | 141.14 | 2.15 |
| Ecoli. | Abyss | 2D | 1 | 170.12 | 2.24 | 170.32 | 2.24 |
| Ecoli. | celera | 2D | 0.25 | 0.11 | 0.96 | 0.1 | 1 |
| Ecoli. | celera | 2D | 0.5 | 0.11 | 0.96 | 0.1 | 1 |
| Ecoli. | celera | 2D | 0.75 | 0.09 | 1.05 | 0.07 | 1.16 |
| Ecoli. | celera | 2D | 1 | 0.11 | 0.96 | 0.1 | 1 |
| Ecoli. | SSAKE | 2D | 0.25 | 2801 | 3.45 | 2797 | 3.45 |
| Ecoli. | SSAKE | 2D | 0.5 | 2878.75 | 3.46 | 2875 | 3.46 |
| Ecoli. | SSAKE | 2D | 0.75 | 2813.14 | 3.45 | 2808.24 | 3.45 |
| Ecoli. | SSAKE | 2D | 1 | 11635.44 | 4.07 | 2765 | 3.45 |
| yeast | Velvet | 2D | 0.25 | 62.12 | 1.8 | 61.97 | 1.8 |
| yeast | Velvet | 2D | 0.5 | 50.87 | 1.71 | 50.74 | 1.71 |
| yeast | Velvet | 2D | 0.75 | 45.9 | 1.67 | 46.2 | 1.67 |
| yeast | Velvet | 2D | 1 | 48.27 | 1.69 | 48.12 | 1.69 |
| yeast | Abyss | 2D | 0.25 | 146.04 | 2.17 | 143.5 | 2.16 |
| yeast | Abyss | 2D | 0.5 | 138.1 | 2.15 | 137.89 | 2.14 |
| yeast | Abyss | 2D | 0.75 | 135.93 | 2.14 | 133.64 | 2.13 |
| yeast | Abyss | 2D | 1 | 124.67 | 2.1 | 117.86 | 2.08 |
| yeast | celera | 2D | 0.25 | 0.11 | 0.96 | 0.1 | 1 |
| yeast | celera | 2D | 0.5 | 0.12 | 0.93 | 0.11 | 0.96 |
| yeast | celera | 2D | 0.75 | 0.12 | 0.93 | 0.12 | 0.93 |
| yeast | celera | 2D | 1 | 0.11 | 0.96 | 0.1 | 1 |
| yeast | SSAKE | 2D | 0.25 | 1398 | 3.15 | 1395 | 3.15 |
| yeast | SSAKE | 2D | 0.5 | 1395 | 3.15 | 1392 | 3.15 |
| yeast | SSAKE | 2D | 0.75 | 1474 | 3.17 | 1471 | 3.17 |
| yeast | SSAKE | 2D | 1 | 1175.8 | 3.08 | 1174 | 3.07 |
